# Olfactory disfunction and diabetic complications in type 2 diabetic patients: a pilot study

**DOI:** 10.1007/s12020-021-02897-6

**Published:** 2021-10-09

**Authors:** Francesco Mozzanica, Anna Ferrulli, Stela Vujosevic, Alessandro Montuori, Arianna Cardella, Andrea Preti, Federico Ambrogi, Antonio Schindler, Ileana Terruzzi, Francesco Ottaviani, Livio Luzi

**Affiliations:** 1 Department of Otorhinolaryngology, IRCCS Multimedica, Milan, Italy; 2 Department of Clinical Sciences and Community Health, University of Milan, Milan, Italy; 3 Department of Endocrinology, Nutrition and Metabolic Diseases, IRCCS Multimedica, Milan, Italy; 4 Department of Biomedical Sciences for Health, University of Milan, Milan, Italy; 5 Department of Biomedical, Surgical and Dental Sciences, University of Milan, Milan, Italy; 6 Eye Clinic, IRCCS MultiMedica, Milan, Italy; 7 Department of Biomedical and Clinical sciences “L. Sacco”, Luigi Sacco University Hospital, University of Milan, Milan, Italy

**Keywords:** Diabetic complication, Diabetes, Olfaction

## Abstract

**Purpose:**

Scarce information on the prevalence and characteristics of olfactory disfunction (OD) in type 2 diabetic (T2D) patients are available. The aims of this study were (1) to assess the olfactory function in T2D patients and to compare it with a control group of individuals without T2D, and (2) to evaluate the differences in OD within T2D patients according to the presence of diabetic complications.

**Methods:**

A group of 39 T2D patients and a control group of 39 healthy individuals were enrolled. Each subject underwent an evaluation of the olfactory performance using the Sniffing Olfactory Screening Test (SOST) and completed a questionnaire assessing the subjective perception of olfaction. According to the presence of diabetic complications, the group of T2D patients was divided into two subgroups. Non-parametric tests and regression analysis were used for statistical analysis.

**Results:**

No differences in the subjective perception of olfaction were demonstrated among T2D patients (with and without complications) and controls. A significant difference for the SOST score was demonstrated among the different groups. In particular, OD was more frequent in T2D patients than in controls. In addition, OD was far more frequent in T2D patients with complications. Regression analysis did not demonstrate any significant association between OD and clinical/demographic characteristics of T2D patients.

**Conclusion:**

T2D patients were more frequently affected by OD. The subgroup analysis suggested a possible relationship between OD and diabetic complications since patients with T2D diabetic complications demonstrated lower olfactory abilities than controls subjects and T2D patients without diabetic complications.

## Introduction

Olfactory disfunction (OD) is defined as the reduced or distorted ability to smell during sniffing or eating. It can be classified as either quantitative, involving alteration in the strength but not in the quality of odors’ perception, or qualitative, in which the quality of odors’ perception is changed [1]. OD can affect up to one-fifth of the general population [2] and about the 60% of individuals over 65 years [3]. The high prevalence of OD is not surprising since numerous reasons could determine an impairment of olfaction, including congenital causes (such as an hypoplastic or aplastic olfactory bulb) [4, 5] and acquired ones, such as trauma; drugs or toxins exposure; age-related impaired ability to regenerate olfactory neurons; nasal obstruction; upper airways infections; psychiatric and neurological diseases [6–11].

Even if supported by only a limited number of previous studies, also diabetes seems to play a role in the development of OD [12–19]. In particular, Kim et al. [19] in a recent systematic review reported that the odds of having OD in patients with type 2 diabetes (T2D) was 1.58 times more likely than in control subjects. Several mechanisms have been hypothesized to play a role in the genesis of OD in T2D patients (including the impact of drugs, metabolism alterations, hypoxemia, oxidative stress, central diabetic neuropathy, hyperglycemia, insulin resistance, and micro- and macrovascular diseases) [20]; however, the results reported so far are inconsistent [21–25]. In addition, the relationship between diabetic complications and OD is still controversial and further evaluative studies are needed.

The aims of this pilot study were (1) to assess the olfactory function in T2D patients using a set of objective and subjective validated instruments in order to evaluate the prevalence of OD in this population, and to compare it with a control group of individuals without diabetes but comparable for sex, age, socio-demographic characteristics, and morbidities (except for diabetes); and (2) to evaluate the differences in OD within T2D patients according to the presence of diabetic complications.

The relevance of this study lies in the fact that a deeper knowledge of the relationship between OD, T2D, and the presence of diabetic complications might be useful in the clinical practice since OD might be associated with a noticeable reduction in quality of life. In fact, olfaction plays a pivotal role for food selection, social communication and harm avoidance. In addition, OD could also interfere with metabolic control because of the changes in dietary habits and/or desire for certain foods that accompany altered olfaction [20]. Finally, a better understanding of the associations between diabetic complications and OD might be helpful to understand the underlying mechanisms for the development OD in diabetes.

## Materials and methods

### Study population

In order to evaluate the prevalence of OD in T2D patients and the association between OD and diabetic complications, a group of 39 T2D patients with a disease duration of more than 5 years and a control group composed by 39 healthy individuals without T2D and comparable for sex, age, socio-demographic characteristics, and morbidities were enrolled. Exclusion criteria for both groups were age over 65 years, use of drugs affecting nasal mucosa; previous trauma of the head; congenital abnormalities of facial growth; systemic granulomatous disease; known mucociliary clearance disorders; known head and neck malignancies; history of previous radiotherapy to the head and neck; previous nasal surgery; cognitive function deterioration (assessed using the Mini Mental State Examination, MMSE [26]); history of alcoholism; endocrine disorders; presence of major depression or an anxiety disorder; inability to give informed consent; pathologies potentially affecting the sense of smell (such as COVID-19 infection, acute or chronic sinusitis). As far as the COVID-19 infection is concerned, subjects with a laboratory-confirmed infection (reverse transcription polymerase chain reaction, RT-PCR), as well as those who reported an history (starting from the beginning of the pandemic) of COVID-19 infection or symptoms known to be highly prevalent in COVID-19 infection (OD, gustatory dysfunction, fever, cough, dyspnea, sputum production, myalgia, arthralgia, headache, diarrhea, rhinorrhea, and sore throat [27]) were excluded. Additional exclusion criteria for the T2D group were fasting serum glucose ≤126 mg/dL; oral glucose tolerance test ≤200 mg/dL; measured glycated hemoglobin (HbA1c) levels ≤6.5%; decompensated diabetes; use of antidiabetic drugs different from metformin or dypeptidyl peptidase-4 (DPP-4) inhibitors; diabetic ketoacidosis at enrollment [12]; variations in anti-diabetic therapy in the 3 months prior to the enrollment.

The data were gathered from the results of the multidisciplinary evaluation of T2D patients which represent the standard of care in our institution. The study was conducted following the principles stated in the Declaration of Helsinki. Informed consent was obtained before the initiation of the study by all the enrolled individuals.

### Olfactory and otorhinolaryngological examination

Each of the enrolled subjects (T2D patients and controls) underwent an otorhinolaryngological (ENT) examination which included a nasal endoscopy (performed with a 30 degrees endoscope, 2.7 mm of diameter), the evaluation of olfactory performance, and the collection of self-assessed evaluations of olfactory function. Nasal endoscopy was performed in order to assess olfactory cleft patency and exclude factors contributing to OD including, for example, acute or chronic rhinosinusitis, or nasal polyps. Olfactory performance was rated using the Sniffing Olfactory Screening Test (SOST, Burghart Messtechnik GmbH, Germany). This test has been validated in various countries and population, including Italy [28]. The SOST evaluates the odor identification abilities using 12 common odorants, each with a list of four verbal descriptors in a multiple forced-choice format (three distractors and one item describing the target odorant). The correct answers are added together and the final score classifies subjects into three groups: normosmic, hyposmic, and anosmic [29]. During olfactory evaluation, the stimuli were presented birhinally about 5 cm under the participant’s nose with an inter-stimulus interval of at least 20 s to avoid adaptation. For subjective evaluation of olfactory function, the Italian version of the Importance of Olfaction Questionnaire (IOQ) [30] was used. It consists of 18 four-scaled items, formulated as a personal statement, divided into three subscales: association (reflecting the emotions, memories, and evaluations that are triggered by the sense of smell), application (analyzing how much a person uses the sense of smell in daily life), and consequence (focused on the importance of the sense of smell in daily decisions). Lower scores reflect poorer olfactory-related quality of life. This questionnaire demonstrated a good internal consistency [30] and it was selected because of its quick administration.

In addition, each patient with T2D underwent:

- Diabetologic evaluation, which included the recording of demographic characteristics and detailed medical history. In addition, blood samples were taken for the measurement of lipids, HbA1c, and glucose. Body weight and height were measured and recorded. Body mass index (BMI) was computed as weight in kilograms divided by height in meters squared.
- Complete physical examination including screening for neuropathy (evaluation of overall muscle strength and tone, tendon reflexes, sensitivity to touch and vibration, through filament test and sensory testing), and screening for peripheral artery disease (pulses evaluation in the legs and feet, measurement of the leg blood pressure taken at the ankle level).
- Ophthalmologic examination to determine signs of diabetic retinopathy.
- Cardiologic examination including electrocardiography, ultrasonography of supra-aortic trunks, and echocardiography in order to identify possible signs of coronary artery disease.
- Neurologic examination including test for autonomic neuropathy (Test Lying to standing performed by photoplethysmographic detector) in order to determine the presence of motor/sensitive peripheral neuropathy.
- Nephrologic examination including urinalysis for microalbuminuria, creatinine, Glomerular Filtration Rate, in order to evaluate the presence of nephropathy.
- Dental examination in order to evaluate the presence of parodontitis or other odontogenic infections.

According to the presence of diabetic complications, the cohort of T2D patients was divided into two groups: Group 1: patients without diabetic complications, and Group 2: patients affected by at least one diabetic complication.

### Statistical analysis

Data were presented as median and confidence interval (CI) for continuous variables or frequency and percentage for categorical variables. The normality of the distribution and the equality of variances were preliminarily tested using Kolmogorov–Smirnov’s test and Levene’s test, respectively. Since a not-normal distribution was found, non-parametric tests were used. Continuous variables were compared using the Kruskal–Wallis and Mann–Whitney tests as appropriate. Categorical variables were compared using the Fisher exact test. Fisher exact test was used to evaluate the associations among the presence of T2D (with or without complications) and OD. Odds ratios and their 95% CI were reported. The association between clinical and demographic characteristics with the presence of OD in patients with T2D was evaluated using logistic regression analysis.

All statistical tests were performed using the SPSS Statistics 24.0 software (SPSS Inc, Chicago, IL). In order to control the increased risks of Type 1 errors, due to multiple comparisons assessed with Mann–Whitney and Fisher exact tests, Bonferroni corrections were performed and a more stringent alpha level for each comparison was set (*p* = 0.025).

## Results

A total of 39 T2D patients and 39 control subjects were enrolled. The T2D group was composed by 24 males and 15 females with a median age of 61 years (CI = 55–64), while the control group was composed by 18 males and 21 females with a median age of 59 years (CI = 52–61). Clinical and demographic characteristics of the enrolled individuals are reported in Table 1. No difference in the distribution of sex, smoking habit, alcohol consumption, and allergy, between the two groups was demonstrated at the Fisher test. Among T2D patients the median duration of T2D was 8 years (IC: 5.1–11.5 years). The presence of diabetic complications was detected in 17 patients out of 39 (43.6%). In particular, nephropathy was the complication most frequently encountered (9/17 cases), followed by macroangiopathy (7/15 cases), neuropathy (5/15 cases), and retinopathy (3/17 cases). Five patients suffered from two or more diabetic complications.

**Table 1:** clinical and demographical characteristics of the enrolled individuals

	Patients with type 2 diabetes (*n* = 39)	Control subjects (*n* = 39)	*p*
Age	61 years (55–64 years)	59 years (52–61 years)	0.187
Sex			
Males	24	18	0.256
Females	15	21	
Smoke			
Non-smokers	31	30	0.876
Smokers	8	9	
Alcohol			
Never/occasionally	37	35	0.726
Habitually	2	4	
Allergy			
None	28	30	0.796
Respiratory	8	6	
Medication	2	2	
Food	1	1	
Diabetic complications			
None	22	/	/
Nephropathy	9		
Macroangiopathy	7		
Neuropathy	5		
Retinopathy	3		

The results are reported as median and confidence interval (in brackets). The results of Mann–Whitney and Fisher tests are reported

### Olfactory assessment

**Fig. 1:**
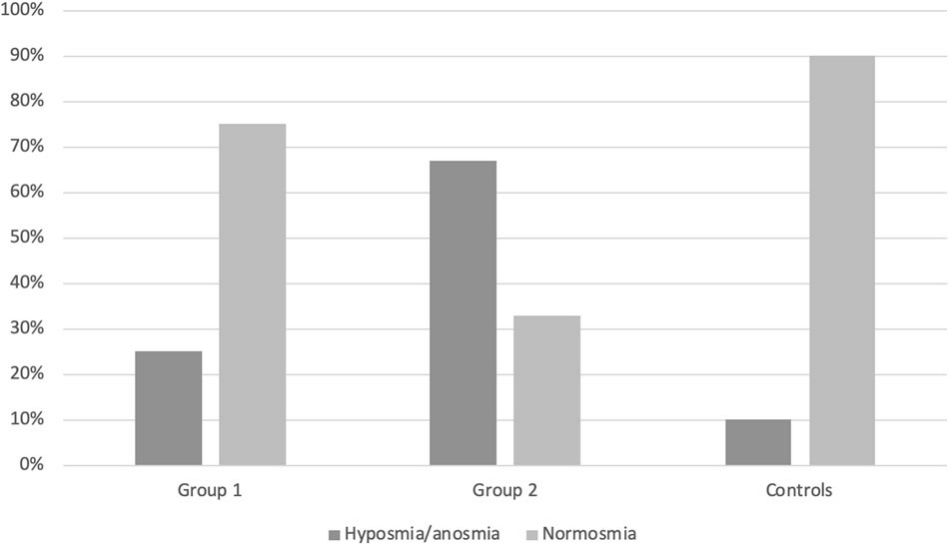
Distribution (in percentage) of OD in the studied population. Group 1 = T2D patients without diabetic complications; Group 2 = T2D patients with diabetic complications; Controls: control subjects

Subjective and objective olfactory evaluation using the IOQ and the SOST respectively was performed in all the enrolled individuals. The results of IOQ and SOST are reported in Table 2. No significant difference was demonstrated for the IOQ score among T2D patients with and without complications and control subjects (*p* = 0.742 at Kruskal–Wallis test). On the other hand, a significant difference for the SOST score was demonstrated among the different groups of individuals (*p* = 0.002 at Kruskal–Wallis test). In particular, the median SOST score obtained in the group of T2D patients with diabetic complications was significantly lower than the score obtained in T2D patients without complications and in the control subjects (*p* = 0.014 and *p* = 0.001 at Mann–Whitney post-hoc test, respectively). In addition, no significant difference was demonstrated between the SOST scores obtained in T2D patients without complication and in the control subjects (*p* = 0.718 at Mann–Whitney post-hoc test). According to the SOST results, T2D patients and control subjects were categorized in normosmic and hiposmic/anosmic (Fig. 1). Only 5 out of 39 control subjects (13%) were hyposmic/anosmic, while 18 out of 39 T2D patients (46%) were hyposmic/anosmic. Patients with T2D were significantly more at risk of developing hyposmia/anosmia than control subjects (OR = 5.829; CI: 1.995–19.834; *p* = 0.002 at Fisher test). When considering only T2D patients, hyposmia/anosmia was far more frequent among patients with complications (13 out of 17, 76.5%) than in those without complications (5 out of 22, 22.7%). T2D patients with complications were significantly at higher risk of developing hyposmia/anosmia than those without complications (OR = 8.319; CI: 2.112–38.841; *p* = 0.001).

**Table 2:** Sniffing olfactory screening test (SOST) and importance of olfaction questionnaire (IOQ) results in the two groups of patients with type 2 diabetes (with and without diabetic complications) and controls

	Patients with type 2 diabetes		Control subjects	*p*
	Without complications	With complication		
SOST	10 (9.25–11)	9 (8–10)	11 (10–11)	**0.001**
IOQ	43 (35.75–50)	44 (40–50)	43 (41–44)	0.742

Median and interquartile ranges are reported (in brackets). The results of Kruskal–Wallis test are reported. Significant differences are highlighted in bold

### Associations among clinical and demographic characteristics and OD in T2D patients

The associations among clinical and demographic characteristics in patients with T2D and the presence of OD are reported in Table 3. None of the analyzed variables was significantly associated with the presence of OD at the regression analysis.

**Table 3:** Association among olfactory disfunction (OD) and clinical and demographical characteristics of T2D patients

	OR	*p*
Age	0.139	1.078
Diabetes duration	0.277	1.060
Allergy	0.962	0.956
Smoke	2.308	0.305
Alcohol	0.911	1.176
Sex	0.478	1.600
HbA1c	1.037	0.147
Glucose	0.998	0.856
Cholesterol	0.985	0.118
Triglycerides	1.011	0.892
LDL	0.990	0.352
HDL	0.967	0.236
BMI	0.975	0.624

The odds ratio (OR) and the results of logistic regression analysis are reported

*HbA1c,* glycated hemoglobin; *LDL-C,* low-density lipoprotein cholesterol; *HDL‐C,* high‐density lipoprotein cholesterol; *BMI,* body mass index

## Discussion

The underlying hypotheses of this article were as follows: (1) OD is more frequent in patients with diabetes than in subjects without diabetes; (2) patients with diabetic complications are more prone to develop OD than patients without complications. In order to confirm these hypotheses the prevalence of OD in T2D patients and the associations among OD, presence of diabetic complications, clinical and demographic characteristics were analyzed. Only few studies analyzed the prevalence of OD in patients with diabetes [12–19] and in none of the previous reports the objective and subjective evaluation of the olfactory function were performed at the same time. The results here reported appear interesting and further suggest a role of diabetes in the development of OD.

### Prevalence of OD

The prevalence of OD was found higher in T2D patients than in control subjects. In particular, it exceeded 45% in the former group, while it was 13% in the latter. This datum is in line with those previously reported [13, 14, 16–18, 23–25, 31]. Falkowski et al. [13], who analyzed olfactory function in T2D patients and controls, found that hyposmia was significantly more frequent in the former group. Le Floch et al. [17] compared smell recognition in 68 patients with T2D and 30 control subjects without known cause of smell impairment and concluded that smell recognition was impaired in patients with T2D. Mehdizadeh et al. [25] found an OD prevalence of 60% in a group of 30 diabetic patients. Weinstock et al. [23] reported that inability to identify odors was higher in patients with T2D than in control subjects (32.3 vs 20%). Similarly, Gouveri et al. [14], and Brady et al. [31] demonstrated a significant reduction of the ability to identify odors in patients with T2D compared to control subjects.

According to the results of the present study, T2D patients were significantly more at risk of developing hyposmia/anosmia than control subjects with an OR of 5.8. This datum appears higher than those reported in previous studies. Kim et al. [19] who performed a systematic review on OD in T2D patients reported an OR of 1.58, while Bramenson et al. [32] found that the risk of anosmia increased with the presence of diabetes with an OR of 2.6. It is possible that the high OR found in the present study might be related to the low number of enrolled subjects; however, also the relatively low prevalence of OD in control subjects (13%) may have played a role. Previous studies reported higher OD prevalence in the general population. For example, Vennemann et al. [2] and Landis et al. [33] found hyposmia/anosmia in 18% and 16% of individuals, respectively. It must be noted that in both these studies a significant percentage of subjects was composed by active smokers and by individuals older than 65 years (factors known to influence olfactory abilities [1, 34]), while in the present study the prevalence of active smokers was low and subjects over 65 years old were intentionally excluded.

### Olfactory-related quality of life

As far as the olfactory-related quality of life is concerned, despite the higher prevalence of OD in T2D patients compared to control subjects, no significant differences in the IOQ scores between these two groups was demonstrated. In addition, among T2D patients, no differences in the IOQ scores obtained by patients with or without complications were demonstrated, even if the prevalence of OD was significantly higher in the latter group. These results are in line with previous reports [35]. In particular, Bramenson et al. [32] found no relationship between impaired olfaction and self-reported diabetes. The discrepancy between self-assessed (subjective) and objective evaluation of olfactory ability might be related to unawareness of olfaction loss that is not uncommon, since olfactory information is processed unconsciously to a relatively large extent [34].

### Effect of diabetic complications on OD

When comparing the OD between T2D patients with and without diabetic complications, hyposmia/anosmia was far more frequent among patients with complications. Only few studies analyzed the association between diabetic complications and OD and the results reported so far are inconsistent. Weinstock et al. [23] and Le Floch et al. [17] found an association between OD and micro- and macrovascular complications. Duda-Sobczak et al. [18] found lower olfactory abilities in diabetic patients with neuropathy and retinopathy. Heckman et al. [36] demonstrated that odor identification was related to polyneuropathy severity. In contrast, other authors did not find any association between OD and diabetes complications. Naka et al. [24] did not find any difference in OD among patients with complicated diabetes, patients with uncomplicated diabetes, and controls. Mehdizadeh et al. [25] did not find any associations between olfactory disturbances and any of the chronic diabetes complications. Moreover, Kaya et al. [37] did not detect any significant differences in olfactory abilities between T2D patients with (nephropathy, retinopathy, and microalbuminuria) and without diabetic complications.

In the present study, T2D patients with diabetic complications were significantly at higher risk of developing OD than T2D patients without complications. In addition, when comparing the prevalence of OD in control subjects, T2D patients with and without complications, no significant differences were found between T2D patients without complications and controls. Similarly, Brady et al. [31] reported that patients with uncomplicated diabetes had no significant differences in olfactory performance when compared with control participants. These results suggest that the presence of diabetic complications is somewhat related to the presence of OD. Since onset and progression of diabetes complications are strongly linked to dysglycemia [20], it is possible to speculate that the same mechanism might also play a role in the development of OD. Recent data suggest a significant association between smelling capacity reduction and increased insulin resistance [38]. As far as the development of OD is concerned, the negative impact of diabetes on olfaction might be related to the impairment of the olfactory receptors (OR) system. This hypothesis is supported by the fact that OR system seems to regulate glucose-stimulated insulin secretion in pancreatic β cells [39]. The role of OR in these physiological processes is explained by the fact that G-protein-coupled receptor subfamilies of OR are present in many non-olfactory tissues such as liver, pancreas, heart, and gastrointestinal tract. Recent evidences show that intestinal OR are able to recognize nutrients such as short, medium, and branched chain fatty acids and to modulate glucagon-like-peptide-1 (GLP-1) and serotonin signaling by specialized enteroendocrine cells [40]. Similarly, taste receptors for bitter substances and free fatty acids are present in the gut and seem to exert an important role in glucose-dependent insulin secretion by modulating GLP-1 [41, 42]. Consequently, OR machinery appears to act as a chemo-sensor of environmental food-derived substances, capable of driving receptor expressions, post-receptor information, cell signaling, and activity in such a way as to modulate metabolic homeostasis in a cell/tissue-autonomous manner. This underlines the enormous potential of the chemosensory signaling of odorant receptors in T2D. However, it must be noted that other authors hypothesized different mechanisms in the genesis of OD in patients with diabetes. In particular, Bitter et al. [21], analyzed the role of hyperglycemia in the genesis of OD and concluded that hyperglycemia, by determining an increased cortex thinning in the insular, cingulate, and orbitofrontal cortex, may facilitate the development of OD [12, 21]. In addition, Lacroix et al. [22], suggested a possible role of insulin in the olfactory mucosa physiology because it improves olfactory sensitivity and discrimination.

### Associations among clinical and demographic characteristics and OD in T2D patients

No significant associations among clinical/demographic characteristics and OD in T2D patients were highlighted using logistic regression analysis. Similar results were reported in previous studies. Yazla et al. [15] found no associations between SOST scores and age, HbA1c, BMI, serum lipids, and diabetes duration. Serraj et al. [16] did not find significant associations between olfactory threshold and sex, age, level of glycemic control, and treatment duration. Gouvieri et al. [14] reported no association between OD and BMI, diabetes duration, and HbA1c. On the other hand, Weinstock et al. [23] found that the ability to identify odorants was strongly predicted by an increasing age, while no associations were detected with glycaemic control, type/duration of diabetes, and diabetes microvascular complications. It must be noted, that in the study of Winstock et al. [23] both patients with type 1 and type 2 diabetes were included and consequently these results appear difficult to compare.

### Study limitations

This study has several limitations. First of all, the number of enrolled individuals is quite small. For this reason, the results here reported should be considered as preliminary and larger studies are needed to confirm these data. In addition, a larger sample size would allow to analyze the effect of confounders of the olfactory function, such as the medications. Second, the instrument used to evaluate the OD comprises only 12 different odorants and was able to differentiate between normosmic and hyposmic/anosmic patients. It is possible that a more detailed olfactory examination might be able to better differentiate the OD characteristics between T2D patients with and without complications and control subjects. However, the SOST is the only instrumented validated in the Italian context. Third, no regression analysis aimed at evaluate the associations among the different types of diabetic complications and OD was performed because of the small number of T2D patients with diabetic complications. Fourth, our sample size was not based on a predetermined power analysis. Larger studies are needed in order to evaluate which diabetic complication is more associated with the presence of OD. Finally, we excluded subjects with a laboratory-confirmed COVID-19 infection, as well as those who reported a recent history of COVID-infection or symptoms known to be highly prevalent in patients with this infection [27]. A more rigorous subject’s selection including laboratory testing with both RT-PCR on pharyngeal/nasal swab and antigen test on blood sample would have increased the strength of the study and consequently the results here reported should be considered with caution. However, it is possible that by excluding subjects on the basis of their past symptoms the risk of enrolling patients with previous or present COVID-19 infection was, at least partially, reduced.

### Conclusion

T2D patients were subject to reduced odor identification abilities. In addition, the subgroup analysis suggested the possibility of a contributory role played by the presence of diabetic complications.

Considering that validated measures of olfactory abilities are readily available as a quick and inexpensive clinical tool, olfactory testing in these patients might be useful for early identification of T2D patients with OD [20]. In addition, since has OD been used as a preclinical indicator to predict the development and onset of diseases (such as Parkinson’s and Alzheimer’s diseases [11, 42]), it is possible that screening for olfactory dysfunction could serve as an early detector for the presence of diabetic complications [20], thus allowing for their prompt recognition and treatment with consequent patient’s benefit. Further studies are needed to explore this hypothesis.

## Data Availability

Data will be available upon request.
